# Reflecting on ICN2: was it a game changer?

**DOI:** 10.1186/s13690-015-0091-y

**Published:** 2015-10-12

**Authors:** Hannah Brinsden, Tim Lang

**Affiliations:** Centre for Food Policy, City University London, Northampton Square, London, EC1V 0HB UK; World Obesity Federation, Charles Darwin 2, 107 Gray’s Inn Road, London, WC1X 8TZ UK

**Keywords:** Nutrition, Food, Sustainability, Public health, Public policy

## Abstract

At the Second International Conference on Nutrition (ICN2), November 2014, 170 member states endorsed the Rome Declaration on Nutrition and a Framework for Action. The Rome Declaration committed to ending malnutrition in all its forms while the Framework for Action offered 60 voluntary actions to help achieve this. These documents and ICN2 itself had the potential to be a major step forward for public health nutrition, addressing issues associated with today’s complex food system. This article reviews ICN2, its process, outputs and some of the gaps and weaknesses of the documents. ICN2’s legacy can be interpreted in two ways–a missed opportunity or one of broad aspirations which have yet to translate into meaningful action. The paper considers whether ICN2 could have adopted a more ecological approach to diet and nutrition, linking health and sustainability. While this fits the evidence, it would require a strong commitment to coherence and food system change, almost certainly a firm stance on some food corporate power, and resolve to champion health at the heart of economic policy. This ambitious agenda would require specific multi-actor and multi-level action, together with metrics and mechanisms for accountability. Coherent government policies and actions to tackle all manifestations of inappropriate diet, and to reframe the economic forces which shape such diets are urgently required. To achieve this, the public health movement needs to work closely with civil society, yet ICN2 showed that there is some reluctance to energise that combination. As a result, ICN2 must be judged a missed opportunity, despite having useful aspirations.

## Background

In November 2014 the second International Conference on Nutrition (ICN2) took place in Rome [1]. This was a much anticipated joint conference of two agencies of the United Nations–the Food and Agriculture Organization (FAO) and the World Health Organization (WHO)–and followed the first International Conference on Nutrition (ICN) held 22 years earlier in 1992. The scope of the meeting was to address malnutrition in all its forms and to improve nutrition throughout the entire life course (See Table 1).

**Table 1:** ICN2 scope and objectives

The key objectives of ICN2 were to:
1. Review progress made since the 1992 ICN including country-level achievements in scaling up nutrition through direct nutrition interventions and nutrition-enhancing policies and programmes;
2. Review relevant policies and institutions on agriculture, fisheries, health, trade, consumption and social protection to improve nutrition;
3. Strengthen institutional policy coherence and coordination to improve nutrition, and mobilize resources needed to improve nutrition;
4. Strengthen international, including inter-governmental cooperation, to enhance nutrition everywhere, especially in developing countries.
The scope of the conference was:
- Global in perspective, but focused particularly on nutrition challenges in developing countries;
- To address all forms of malnutrition, recognizing the nutrition transition and its consequences;
- To seek to improve nutrition throughout the life cycle, focusing on the poorest and most vulnerable households, and on women, infants and young children in deprived, vulnerable and emergency contexts

Like most UN processes, ICN2 was the culmination of a number of preparatory and consultation phases held between 2011 and 2014. These preparatory meetings brought together experts and stakeholders in the fields of both health and agriculture and were used to prepare technical papers and collate best practice on improving nutrition [2]. The purpose of this long process was to inform what would be the ultimate outputs of ICN2: the Rome Declaration on Nutrition (RDN) and a Framework for Action (FFA) [3, 4]. Alongside the formal process of Member State consultations, a Civil Society Liaison Group was set up and the Global Forum on Food Security and Nutrition (FSN Forum), hosted by the FAO, provided civil society an opportunity to comment and discuss on the early documentation drafts [5].

Through the RDN and FFA, member states committed to eradicating hunger and ending malnutrition in all its forms. The message was familiar, focused on investing in nutrition, co-ordination across sectors, promoting sustainable and coherent food production and consumption, improving nutrition education, empowering people to create healthy environments, provisions for women and mothers, reducing rates of overweight, stunting and wasting and the associated deficiencies, improving nutrition as part of emergency responses and incorporating nutrition into the post-2015 agenda. Few could criticise these goals.

## Discussion

As the immediacy of ICN2 fades questions remain over whether or not ICN2 made a difference. Do the documents sufficiently challenge the attributes of modern food systems which are widely recognised as failing to deliver what is needed? Do they identify actions capable of shifting the current food policy paradigm towards something in which public health and eco-systems viability are at the heart of food production and consumption? Do the documents mandate action or provide clear targets? As outlined in Table 2, the answer is mixed. Laudable aspirations may be there, but the contents of the documents do not appear strong enough or systemic enough to challenge the *status quo*. A year after the events, it is hard to see how ICN2 can be considered a ‘game changer’ and to have transformed global food and nutrition politics or, more specifically, to have operationalized better links of food and nutrition interventions with the range of other pressing issues suggested by the evidence on food systems. These include food’s environmental challenge [6, 7],–not least climate change whose policy structures were to be decided by the end of 2015 [8]. Food is and remains a major cause of environmental damage, but it is also the terrain on which much wider politics is fought out: urbanisation, employment, distribution of wealth, social inequalities, the nature of progress and modernity. All these attributes of modern food systems could and should be central to any debate about the future but despite copious evidence on the harshness of food markets, the legacy of social divisions within and between societies, the threat of impending climate change and ecosystem loss, full and frank debate was largely side-lined or de-emphasised over the ICN2 processes. So must ICN2 be judged for its timidity rather than silence? Is its failure to re-energise food debate after a moment of international interest in and immediately after the Great Recession of 2007–10 a missed opportunity rather than a failure of evidence, a lack of leadership or the inevitable dilution of vast multilateral processes?

**Table 2:** Was ICN2 a ‘game changer’?

Yes–the following key issues were recognised	No–the following key issues were side-lined or ignored
The concept of ‘malnutrition in all its forms’ introduced, rather than dealing with under- and over-nutrition in silos	Upstream drivers of malnutrition
The global burden of obesity was recognised as a problem, even in developing countries	The environmental challenge posed by the current food system
The mismatch of economics and health recognised	The burden and challenge of climate change and the impact of food systems on this
The need for investment in nutrition	Social divisions within food systems and inequalities of food markets
The need for cross-sector coordination on issues related to nutrition	Insufficient challenge to the attributes of the food system which are recognised as failing
Promoting sustainable and coherent food production and consumption	The actions specified were agreed but were not mandated
Improving nutrition education	Specific targets were not set to allow clear monitoring or accountability
Empowering people to create healthy environment	
Improving nutrition as part of emergency responses	

Source: authors

To damn ICN2 with faint praise may seem harsh; it is easy to criticise global gatherings as compromised, but let us consider the stark evidence of the need for firm policy reorientation via meetings such as ICN2. If not here, where can this happen in a globalised world? One big change since the first ICN in 1992 is the rapid rise in obesity and the fragmentation of the picture of diet-related ill-health. 0.8 billion people worldwide are undernourished [9], and experience symptoms of wasting and stunting, while more than 1.5 billion adults are overweight or obese [10]. In many countries a triple-burden of malnutrition exists with the co-existence of under-, mal- and over-nutrition. This complexity occurs across and within countries, communities, families and even individual life-courses. While the ICN2 documents acknowledge some of the complexity posed by the co-incidence of under-, mal- and over-nutrition, they stop short of offering viable solutions. The thinking underpinning the documents appeared locked into ‘policy business largely as usual’. Yet, if change is to occur, this paradigm needs to be challenged and replaced. While pragmatists might judge that global gatherings are hardly likely to be game-changers, the critics are nonetheless correct that the world must dispel any fantasy that business-as-usual will deliver results; timidity is part of the problem.

ICN2 certainly witnessed a range of opinion, from realists to radicals, from pragmatists to incrementalists, from advocates of ‘nutrients first’ to ‘food systems change’. But this range of positions was compartmentalised along familiar UN lines: inside the main hall versus outside, rich country versus poorer ones, pro-growth versus sustainability first adherents, and more. These divergences need to be addressed rather than be used as justifications for blocking the dialogue about viability and strategy that urgently needs to happen, if the world is to sort out the slow car-crash that is the food system’s impact on health and environment. At ICN2 this simply did not happen, which was and remains a missed opportunity.

Whatever this missed opportunity was due to, it was not lack of evidence. In this respect ICN2 fits a pattern noted by other analysts and leads to questions as to whether existing UN bodies are appropriate for the enormous challenges ahead. On trade policy, it should be noted, bilateralism is replacing globalism; there has been no revision of world trade rules since 1994 but bilateral deals proliferate, including on food. One review of the failure of international food and health governance, in this case of the failure to eliminate maternal and child undernutrition in high-burden countries, concluded that recurring themes in the failure of transnational institutions were: fragmentation, lack of an evidence base for prioritised action, institutional inertia, and failure to join up with promising developments in parallel sectors [11]. It called for a radical overhaul of institutions. Lack of evidence is not something that can be said to apply to food and health; lack of evidence of successful interventions perhaps but not lack of evidence suggesting the need to try. Critics might argue that in this respect, ICN2 was ‘situation normal’ and showed how mired the UN has become in the competing narratives and interests of post-modern world ideologies and economies; and that the high ideals voiced in the 1940s [12] or 1970s [13] for food system reform [13] have been subsumed in the realpolitik of a world dominated by corporate power where markets rule and economics triumphs over public health [14, 15].

The capacity to chart a clear way forward at ICN2 was not helped by the fact that it was a meeting of not just one but two UN institutions with different mandates, budgets, political traction and priorities, the FAO and WHO. While the WHO focuses on health across systems, throughout the life course and in preparedness and prevention of non-communicable diseases (NCD) and communicable diseases, the FAO is focused on eradicating hunger and malnutrition, eliminating poverty and driving economic progress. While these agendas can align in many ways, the economic solutions to poverty and malnutrition have been largely penetrated by a prevailing market ethos which critics see as favouring deference to multi-national corporations, foreign direct investment and a processed food paradigm of which health researchers are increasingly critical . The failures of ICN2 therefore go beyond the normal failures of the UN system to a systemic failure of the ‘wicked problems’ linked to the disconnect between those focused on eradicating hunger and poverty and those focused on promoting health [16].

That said, ICN2 gave some grounds for optimism. The societal awareness of the enormity of the food and health challenge is spreading. Worries about the thoughtless spread of meat-based diets grows [17]. Even poor countries now acknowledge rising obesity. Concern about the tsunami of ‘non-food’ foods washing over the world and distorting diets also grows [18], fuelled by experience of the wiles of marketing [19]. At the policy level, even a few years ago, the mismatch of economics and health was dismissed and the default position was that health follows wealth. The 2007–08 commodity and banking crisis momentarily dented the confidence of market advocates, but they quickly reasserted their version of normality. Its allure, however, is less assured inside the World Bank and the IMF. The latter, for instance, now recognises that stark inequalities dampen growth. Rightly, policy advisors are quietly moving away from the fiction that vast inequalities can be justified by trickle-down economics [20, 21].

The agenda laid out by ICN2 must be judged in this more fluid context; it is only one global convention amidst many. But that, surely, is why there was no need for Ministers of Health to be cautious. On the contrary, now is the time to argue fiercely that health should be at the heart of sustainable development, to be central rather than a ‘bolt-on extra’. Surely the state of food and nutrition today can be transformed only if reshaped also to meet the sustainability agenda (low carbon, low water, supporting biodiversity, waste-reducing, land use efficient, etc.), by being firm about (rather than kowtowing to) food corporate power and by insisting that health is at the heart of economic policy. This combination poses enormous challenges and is not for the faint-hearted, but it is what the evidence suggests is needed for the mid-21st century. And what else are meetings such as ICN2 if not for making such commitments? ICN2 was a golden opportunity to inject these perspectives into national and international public discourse. Here is where the imagination for change could be sparked.

## Characteristics for future food systems: sustainability not just health

Post ICN2, even more than before, a serious debate is needed about how, and how extensively, the food system can be transformed. Can hunger, malnutrition and obesity be resolved by minor modifications of existing food systems? Most analysts think not [22]. Multiple change at multiple levels is required. While claiming to present a new framework for responding to modern nutrition challenges, the ICN2 FFA emphasised the role of supplemented feeds for treating deficiencies, on ensuring adequate feeding, on managing obesity and on education. This resort to ‘technical fixes’ is implausible and unlikely to ‘end malnutrition in all its forms’. This is an old-style policy reductionism inappropriate for complex problems; it does not address the source of problems. The radical thinking among food analysts lies more in how to create food systems delivering diets based on minimally processed foods, how to normalise sustainable diets–ones which blend public and eco-systems health–from sustainable (i.e. low carbon, low water, high skill, high nutrient) food systems [23, 24]. As obesity and other health conditions grip middle income countries [25, 26], surely ICN2 ought to have focussed on how this could be prevented, not simply managed and treated. An ecological public health perspective proposes that policy deliberations should centre on how to create the conditions for good health, and how to tackle the wider economic and socio-cultural determinants of health on which evidence has emerged so strongly since the first ICN in 1992 [27, 28].

To support such change, policy makers need to achieve a ‘health grip’ on the food economy, something that history teaches us is never easy [29]. But crises are occasions when public health considerations, if firmly championed, can win a place at the policy high table. The case for doing this is clear, and is what ICN2 ought to have articulated more strongly. The cost of diet-related ill-health is vast, with healthcare services paying for the excessive space food companies have been given in the name of liberalised trade. During ICN2, the McKinsey Global Institute released a report estimating obesity now costs an annual $1.2 trillion [30]. Two weeks later, a Wellcome Trust and WHO funded study of antimicrobial resistance (AMR)–a headache now becoming a nightmare exposing how technical fixes can be squandered–estimated AMR will cost a cumulative 300 million lost lives and £100.2 trillion lost to global GDP in 2014–50 [31]. Food and farming are key users and abusers of antimicrobials. Such enormous sums now suggest that the pursuit of ‘cheap’ and over-produced commoditised food, the goal of the 1940s, is too crude and in part actually a dangerous goal. The vast externalised costs and the distortions of human and planetary health through the current food system ought to have been the refrain at ICN2, posing an unprecedented threat to developing nations and energising new direction of food supply chains, better eating patterns, more vigorous controls over the drivers of diet-related ill-health. These ought to be a feature of the ‘health in all policies’ transition from the Millennium Development Goals to the Sustainable Development Goals [32].

## Governments need to be more specific in their commitments

Addressing malnutrition requires formidable political will, and specific and binding agreements alongside the shared understanding of problems, something that ICN2 failed to deliver. Here lies a distinction between legal statutes which are binding and clear, and policy documents which may be anything from aspirational to addressing minutiae. ICN2’s agenda ought to have been the global agreement to tackle the drivers of poor diets and malnutrition, obesity and inequalities and challenging current norms and committing to work towards a new food system paradigm. The history of public health in general and the pursuit of safe, healthy food in particular shows that this frequently means confronting conflicts of interest and market power [27]. This is why the principles of protecting human rights and creating free and open markets which do not undermine health requires regulation and standards. There are always incentives for commerce to undercut competition and take shortcuts; frameworks which set what is meant by markets are long overdue.

ICN2 was shy about calling for strong intervention to tackle both under- and over-nutrition; the messages were aspirational, not specific enough to allow for accountability an auditing which are essential for change. Is this because academics haven’t helped stiffen the policy sinews of the policy-makers? In some cases, such as on marketing, perhaps, although there has been no shortage of thinking and evidence [33, 34]. But on high carbon, high calorie diets, the work has been voluminous. The evidence of the need to cut carbon emissions from the food system is overwhelming, and is a rising theme for both the SDGs and the 2015 climate change talks [35]. At ICN2, few member states offered specific commitments on what they would actually do to tackle the nutrition challenges. Conversations on action were not entirely absent from the conference, but took place at a series of roundtable discussions held throughout ICN2, where there was agreement that cross-sector action, policy coherence and governance and accountability frameworks were sorely needed. The absence of specific commitments must be addressed at member state level, or ICN2 will go down in history as hollow words.

## Metrics and indicators

To this end, a set of tighter metrics and mechanisms by progress (or lack of) can be monitored is crucial. Whilst the onus of responsibility must lie primarily with governments, other stakeholders must also because more vociferous about the case for system change in whatever way they can, whether this is corporations improving their own supply chain, civil society applying maintaining pressure for action or research institutions gathering data on what works, when and how. Without these pressures on international forums, the broad commitments made in the FFA, the impetus for action, the political will and, above all, the financing of the required specific interventions are likely to be weak. It is widely acknowledged that “what gets measured gets done” and, in the phrase attributed to US World War 2 General Eisenhower, “the uninspected quickly deteriorates”. The shopping list of actions provided in the FFA are not in themselves enough to create the leverage or accountability for actions, nor are they immediately translatable into indicators. In the absence of indicators within the FFA itself, it is necessary to look to existing means of accountability and governance, both within and outside of the UN system, and to harmonize these tools so as to align priorities moving forward. The UN itself has a number of monitoring systems in place, for instance linked to NCDs, maternal nutrition and MDGs, which focus on health outcomes. Additional to these, there are examples of monitoring mechanisms coming from civil society which focus on the policy actions being taken to achieve policy goals. Three such monitoring tools which are of particular relevance to the nutrition agenda discussed at ICN2 are: (1) Food-Epi which has been developed as part of the International Network for Obesity Research, Monitoring and Action Support (INFORMAS) [36], and focuses on the monitoring and benchmarking of government policies related to food environments with the goal to reduce NCDs and obesity; (2) the Hunger and Nutrition Commitment Index (HANCI) [37], which also focuses on government policies but linked to tackling hunger; and (3) the Access to Nutrition Index (ATNI), funded by the Bill and Melinda Gates Foundation and the Wellcome Trust [38] which focuses on the nutrition related policies of the food and beverage industry. Such tools can help assess the progress that is made across different sectors and through benchmarking, ranking and rating can help to stimulate and improve action around the world, and take a more critical approach to assessing action rather than health outcome alone. Monitoring and accountability for actions is absolutely vital and may well be at least one of the mechanisms that supports actual change.

## Addressing concerns of corporate power and accountability

A theme running throughout ICN2 side-meetings concerned the role of the food industry, its power and accountability. There are now decades of experience of ‘partnerships’ and ‘stakeholder inclusiveness’ [39, 40], all framed by broadly neoliberal-inspired belief that the state role should be minimised and replaced by market dynamics. Yet in food systems, this is precisely what predominates and has been found wanting. Corporate responsibility (CR) is a poor substitute for market reframing, but CR has been offered as the route to improved diet action, with companies promising product reformulation as the key to obesity for example. Changing product recipes may be good brand protection but has little population dietary impact, and is no compensation for vast marketing budgets. Some sections of the food industry are ideologically opposed to public health intervention, arguing that it demeans individual choice. They favour a technical approach to nutrition to justify the products that they produce and sell. Improving public health requires food business to change, of course, but the questions are: in what direction, how and how much? The key discussion at some fringe ICN2 meetings was about who sets the terms of reference for change. Evidence on the nutrition transition in the developing world suggests that ‘soft’ neo-liberal appeals to rational consumerism are of limited value. The vast marketing and distribution budgets of ‘Big Food’ mean the relationship between producer and consumer is unequal, not the much-vaunted ‘partnership’ sold to health agencies [41]. Unfortunately, ICN2 missed the opportunity to discuss these issues seriously in the main hall or texts, and thus lost the opportunity to address differences about visions of the future and how to get there.

## Civil society is not only important but necessary life blood

Civil society organisations were much in presence at ICN2, but mostly they left somewhat dejected. They are actually key forces for any transformation of food and health, not least as advocates, scouts and public voices at multi-levels and multi-stages [42–44]. And they have an important role in creating the leverage and political will necessary for change and for challenging issues such as corporate power. Civil society organisations can help provide ‘policy space’ for political leaders to push framework change. At ICN2 it was only relatively late in the long preparatory process that civil society was invited to engage in the ICN2 process, and even that required a long process of negotiation between some leading civil society individuals and the FAO. Thankfully, in the end, civil society was given a place. More than 150 civil society organisations came together, from a wide range of backgrounds, countries and interests, to develop a declaration emphasising the need for meaningful governmental commitment and steps after ICN2 [45]. An open letter was presented to the heads of the WHO and FAO, supported by over 300 individuals, advocacy organisations and academics from around the world [46] which proposed a mechanism similar to the Framework Convention on Tobacco Control (FCTC) for food. This argued that a legal framework and policy coherence free from conflicts of interest is required at a global level if malnutrition in all its forms is actually to be achieved [47].

A consistent theme in and outside the halls for civil society organisations at ICN2 was: what would it take to get ‘step-change’, systems change, framework change? The metaphors varied but the sentiment did not. They felt that government public health agencies were too often on the back foot, in thrall to economic ministries, who are focused on righting the world’s economy by tightening expenditure, even though there is debate about whether this is short-sighted. The case for prevention of diet-related ill-health is surely overwhelming. It ought to be popular yet meets resistance. ICN2 was not therefore the time for health bodies to be deferential but strong; they ought to be helping their populations live under conditions which maximise the chance of healthy lives.

## A decade of nutrition

The FFA called on the United Nations to declare 2016–2025 a ‘Decade of Nutrition’. This deserves strong support, and was endorsed by the 68th World Health Assembly in May 2015. Such a declaration could be a powerful rallying point for change, particularly if it means countries commit to implement specific priorities in a binding or quasi-binding way. A Decade of Nutrition could draw on the FFA, as well as the priorities of other agendas from across the UN-system, such as the WHO Action Plans to tackle NCDs [48] and to improve maternal, infant and child nutrition [49], to help address the issues raised in the present paper in a more comprehensive and harmonized way than the FFA was able to achieve on its own. A Decade of Nutrition could also dovetail with the post-2015 Sustainable Development Goals agenda. A few binding commitments for specific action to address some of the fundamental underlying issues related to malnutrition may well prove to be more powerful than a far larger number of broad and non-binding actions.

## Conclusions

ICN2 could not be expected to resolve all the world’s food and health problems in one short three day meeting. In the middle of World War 2, the free world met for over three weeks to begin to do that [50, 51]. Food and health policy has become much more complex since then. But ICN2 did have the potential to shape future discussions and actions around a reinvigorated public health nutrition. In this it failed. While raising the profile of nutrition on the global agenda, it stopped short of offering a sufficient response to the 21st century challenge–to work out what a good food system could look like and the role that different actors should play. Climate change advocates are already modelling what this means in health and agricultural terms [52, 53]. This will require coherent government policies and actions which concurrently tackle all manifestations of inappropriate diet, addressing consumerism and those forces which shape bad diets. This is a big challenge, but the evidence suggests nothing less is needed. Whether the policy starting point is climate change or health, equality and rights or economic development, food sits at their meeting point. In our view, the policy and political challenge that ICN2 skirted round, yet nonetheless raised, is how public health nutrition fits within sustainable development. We see the case for policy to adopt an ecological approach to diet, nutrition and the creation of low impact food supply chains. Public health advocates must be diplomatic but firm when holding governments and food commercial interests to account, just as they must help the public to resist the charms of cheap processed foods. A mix of multi-sector action, policy coherence and improved governance and accountability mechanisms is needed. We need to end not just “malnutrition in all its forms” but diet-related ill-health in all its forms. The dire and complex state of global diet-related ill health requires nothing less. If ICN2 failed to deliver that, the tensions nevertheless were present. For that we should be grateful. But more, much more, needs to happen.
